# 

**Published:** 1896-03-14

**Authors:** 


**Notices of Births, Deaths, and Marriages** are inserted in this column at a charge of 2s. 6d. for an announcement not exceeding four lines.

**Notice to Advertisers and Subscribers.**

All Advertisements, Orders for Copies of The Hospital and Business Communications should be addressed to **THE MANAGER, (Not to The Editor)**, The Hospital, 428, Strand, London, W.C.

The Cost of Subscription, post free, is as follows:—Per week, 2½d.; per quarter, 2s. 8d.; per half-year, 5s. 3d.; per annum, 10s. 6d. Foreign:—Per Annum, 15s. 2d.; payable, in all cases, in advance.

### NOTICE TO CORRESPONDENTS.

All MS., Letters, Books for Review, and other matters intended for the Editor should be addressed The Editor, The Lodge, Porchester Square, London, W.

The Editor cannot undertake to return rejected MS., even when accompanied by stamped directed envelope.

**"Burdett's Hospitals and Charities."**

Sixth year of publication. 950 pp. Scarlet cloth, gilt lettered. Price 5s. A most complete guide to British, Colonial, Indian, and American Hospitals, Dispensaries, Nursing and Convalescent Institutions, and Asylums. A few complete sets of the preceding five issues of the "Annual" may still be obtained on application to the Publishers, The Scientific Press (Limited), 428, Strand, London, W.C.

**NOTICE.—Vol. XVIII. of "The Hospital" (April to September, 1895), in handsome cloth binding, is on sale at the Office of this Journal. Price 7s. 6d. Cases for binding the Numbers contained in this Volume, 1s. 6d. Index to Vol. XVIII., 2½d., post free.**
**The Monthly Part for March is now ready, price 6d., by post, 8d.**

### SCRAPS AND GLEANINGS.

The annual ball in aid of the Fleming Memorial Hospital was held quite recently.

At the Middlesbrough Fever Hospital 38 patients have been received during the year.

A good balance in hand exists on this year's accounts of the Haywood Hospital, Burslem.

The Duchess of St. Albans presided at the re-opening of the wards of the East London Hospital.

The Chelmsford Infirmary and Dispensary is soon going to add four new wards to the existing building.

The Royal Normal College, Norwood, is to be purchased as a school for the blind children of the metropolis.

Plans for an infectious diseases hospital at Mexborough have been laid before the District Council, and approved of.

An anonymous donor has presented the Grosvenor Hospital for Women and Children with £3,000 towards the building fund.

As compared with last year, the number of patients at the Brighton Dispensary shows an increase of 1,000 patients during the twelve months under review.

The Wantage Cottage Hospital presents a highly creditable report this year, and the income of the hospital shows a considerable increase over any previous year.

An increase in the working men's subscriptions to the Dewsbury and District General Infirmary is shown in this year's accounts, but the annual subscriptions remain stationary.

The Edinburgh Local Government Board has intimated approval of the site and construction at Colington Mains of the hospital which has recently been removed from the Queen's Park.

At the annual meeting of the Kendal Provident Dispensary it was stated that the subscribers' donations had dropped from £60 to about £47. About 2,000 persons benefit through the society annually.

The women of Cardiff have prepared a petition for the Home Secretary, recommending that the successor to the surgical appointment at the prison should be a woman, the present surgeon having resigned.

The annual meeting of the Harrogate Royal Bath Hospital, which took place recently, showed in the report that during 18[illegible]5 there were a total of 1,100 patients, as compared with 1,033 in the previous year.

A cheque has recently been sent to the British Home for Incurables by Messrs. Skeffington and Son, as the result of further sales of Canon Fleming's sermon preached at Sandringham on the occasion of the death of the Duke of Clarence.

At the quarterly board meeting of the Leicester Infirmary the balance at the opening of the present year was shown to have increased to £393. This was principally owing to the proceeds of the Children's League of Love, which amounted to £380.

Owing to the great increase of out-patients at the Glasgow Royal Hospital for Sick Children, the directors have decided to increase the accommodation of the dispensary, and the Bellahouston Trustees have agreed to give £1,000 for this purpose.

The twenty-third annual meeting of the Birmingham Children's Hospital has taken place. The committee are appealing to the sympathy of all subscribers and friends for increased support to the finances of the institution, which are in serious need of help.

The total number of patients helped during the year by the Bradford Infirmary Samaritan Society has been 273. This year's receipts in connection with the fund amounted to £465. Over 870 garments have been distributed among the patients in the last twelve months.

Contributions for the National Colony for Epileptics are being earnestly enlisted. About £1,200 have been received in response to the appeal, a sum which is quite inadequate for the purposes in view, which are the erection of certain much-needed developments of our National Colony.

The directors of the Dumfries and Galloway Royal Infirmary present a highly satisfactory account of the year's workings of their institution. In spite of the fact that an exceptionally large number of cases have been treated in the wards during the year, a fair credit balance remains on the accounts.

The Court of the Worshipful Company of Leathersellers have recently made a grant of ten guineas to the funds of the Rescue Society, 79, Finsbury Pavement, E.C., which is greatly needing help at the present time on behalf of its reformatory and preventive work amongst girls and young women.

At the annual meeting of governors of the Royal Alexandra Hospital for Sick Children at Brighton, the institution was shown to have fully maintained its customary prosperity during the past year. It was decided to erect a nurses' dormitory in the grounds of the hospital, at an estimated cost of £1,200.

The annual report of the Longmore Hospital for Incurables, at Edinburgh, shows that there is now accommodation for 110 patients. The managers hope shortly to be able to provide for even more, owing to a legacy of £25,000 left to the hospital by Mr. Waddele, of Leith. When the new wing is built it is proposed to set apart two wards for phthisical patients.

Some time ago Her Majesty the Queen presented to the County Hospital, Reading, a woollen hood worked by her own hands, which has been kept in one of the children's wards, and is an object of interest both to the inmates and the visitors. Mrs. C. T. Murdoch has presented to the hospital a handsomely-framed portrait of Her Majesty for the same ward. The Duke of Wellington has sent a present of game for the patients of the institution.

The committee of the Liverpool Northern Hospital are appealing to their friends and the inhabitants of Liverpool to assist them in raising the sum of £40,000, in addition to the grant of £10,000 by the Corporation. This amount, by placing the finances of the institution in a sound position and establishing a maintenance fund, would enable the committee to avail themselves of the munificent grant of £[illegible],000 from the David Lewis Trustees for the purpose of building a new hospital and nurses' home.

### BOOKS RECEIVED.

J. AND A. CHURCHILL.

"A Handbook on Leprosy." By S. P. Impey, M.D.

"The Schott Method of the Treatment of Chronic Diseases of the Heart. By W. Bezley Thorne, M.D.

THE SCIENTIFIC PRESS.

"Infant Feeding by Artificial Means." By S. H. Sadler.

JOHN MURRAY.

"With an Ambulance During the Franco-German War." By Charles E. Ryan.

SAMPSON, LOW, AND MARSTON.

"Twentieth Century Practice: An International Encyclopædia of Modern Medical Science, by leading Authorities of Europe and America." Edited by Thos. L. Stedman, M.D., New York. Volume IV.

Reprinted from the EDINBURGH MEDICAL JOURNAL, 1894 and 1895. "On the Diagnosis of Tubercular Joint Disease." By A. G. Miller, F.R.C.S.E.

**Periodicals and Pamphlets.**—English Illustrated Magazine, Marcus Ward's Magazine, Charity Organisation Review, Therapicé, Pediatrics, Columbus Medical Journal, Guy's Hospital Gazette, Medical Press, Invention, Literary Digest, Medical Week, New York Medical, Building News, Whitehall Review, The Humanitarian, Cassell's Family Magazine, Cornhill Magazine, Sunday at Home, Boy's Own Paper, Girl's Own Paper, Leisure Hour, The Zenana, Child's Guardian, The Corpuscle, International Medical Magazine, Clinical Journal, London, Hermes, New York Medical Journal, British and Colonial Druggist, Electrical Review, The Sun, Public Health, Clinical Sketches, Nursing Notes, Practitioner, Photographic Review, Medical Press, Westminster Review, Tri-State Medical Journal, Leeds Hospital Gazette, Medical Bulletin, Monthly Homœopathic Review, Transactions of the Cremation Society of England, Food and Sanitation.

### THE STUDENT OF MEDICINE.

**ROENTGEN'S PHOTOGRAPHY.**

"A CLINICAL CLERK" writes: I am a constant reader of THE HOSPITAL, and I thought that the following might be of some interest to your readers. On Tuesday last (February 11th) Dr. B. Stedman, senior house surgeon at the Sheffield Royal Hospital, had a boy's foot "shadow-graphed" by this new method of Professor Rnoetgen's. The boy was suffering from the effects of a needle which had pierced his foot and broken off short. The "shadow-graph" showed the position of the needle, and the same evening Dr. Stedman cut down upon it and

THE STUDENT OF MEDICINE—*continued.*

removed it, using the "shadow-graph" as his guide. This, I think, is one of the first instances in the provinces of this new process being used in the aid of surgery. The photograph was taken at Firth College, Sheffield.

### VACANCIES.

The following vacancies are announced this week, the amount of salary in each case being given after the name of the institution :—

### Resident Medical Officers.

Bristol General Hospital.—House Surgeon ; doubly qualified. Salary £120 per annum, with board, residence &c., in the house. Appointment for three years. Applications to the Secretary by March 18th.

Cornwall County Lunatic Asylum, Bodmin.—Junior Assistant Medical Officer. Salary £100 a year, increasing £10 yearly to £120, with board, lodging, &c. Applications to the Medical Superintendent at the Asylum by March 19th.

Dr. Steevens's Hospital, Dublin.—House Surgeon. Appointment for two years. Salary £100 per annum, with apartments, fire, and light. Applications to the Governors and Guardians of Dr. Steevens's Hospital, Dublin, by March 21st.

General Infirmary, Leeds.—Resident Surgical Officer. Salary £100 per annum, with board, residence, and washing. Applications to the Secretary of the Faculty by March 20th.

General Hospital, Nottingham.—Assistant House Physician and an Assistant House Surgeon. Board, lodging, and washing provided in the Hospital. No salary. Applications to the Secretary, General Hospital, Nottingham, by March 18th.

Greenwich Union Infirmary.—Second Assistant Medical Officer; unmarried. Salary £80 per annum, with board, lodging, washing, and attendance in the Infirmary, and 2d. per day in lieu of beef. Appointment till March 25th. Applications to Samuel Saw, Clerk to the Guardians, Union Offices, Greenwich, by March 18th.

National Hospital for Consumption for Ireland.—Resident Medical Officer and Registrar. Salary £160 per annum, with apartments, board, &c. Applications to the Honorary Secretary, 37, Dame Street, Dublin, by March 14th.

Victoria Hospital for Consumption and Diseases of the Chest, Edinburgh.—Resident Physician. Board and lodging provided and allowance for conveyance at the rate of £24 per annum. Appointment tenable for six months. Applications to Messrs. Wallace and Guthrie, Honorary Secretaries, 1, North Charlotte Street, Edinburgh, by March 13th.

Victoria Hospital, Folkestone.—House Surgeon. Salary £80 per annum, rising £10 annually to £100, with board, residence, and washing. Applications to the Secretary by March 20th.

West Riding Asylum, Wakefield.—Two Resident Clinical Assistants. Appointments for six months or longer by mutual agreement. No salary, but board and furnished apartments provided. Applications to the Medical Superintendent.

West Sussex Asylum.—Medical Superintendent. Salary £450 a year, with unfurnished house (the Committee paying rates and taxes), light, washing, coals, and vegetables. Applications endorsed "Medical Superintendent" to Ernest H. Blaker, Clerk to the Committee, West Pallant, Chichester, Sussex, by March 25th.

### Non-Resident Medical Officers.

Charing Cross Hospital.—Assistant Surgeon; must be F.R.C.S.Eng. Applications to Arthur E. Reade, Secretary, by March 25th.

Poplar Hospital for Accidents, Blackwall, E.—Honorary Surgeon. Applications to the House-Governor by March 13th.

Victoria Hospital for Consumption and Diseases of the Chest, Edinburgh.—Non-Resident Medical Officer for Out-patients. Salary at the rate of £60 per annum. Appointment tenable for six months. Applications to Messrs. Wallace and Guthrie, Honorary Secretaries, 1, North Charlotte Street, Edinburgh, by March 13th.

Victoria Hospital for Sick Children, Queen's Road, Chelsea, S.W.—Second Honorary Anæsthetist; must be legally qualified. Appointment for one year, subject to annual re-election. Applications to the Secretary by March 21st.

Westminster Hospital, Broad Sanctuary, S.W.—Fourth Assistant Surgeon; must be F.R.C.S.Eng. Applicants to send in certificate of age and attend the House Committee with testimonials on March 17th.

STANDARD TEXT-BOOKS.

Just Out. Second Edition.

**INFANT FEEDING BY ARTIFICIAL MEANS:** A Scientific and Practical Treatise on the Dietetics of Infancy. By S. H. SADLER, Author of "Suggestions to Mothers," "Management of Children," "Education." Second Edition, with a new chapter, and several coloured plates, illustrative of feeding-bottles used in ancient times, &c. Crown 8vo., cloth gilt, with 10 plates and many Illustrations in the text, price 5s. Facsimile Autograph Letters from the late Sir Andrew Clark, M. Pasteur, &c., &c.

"Mrs. Sadler's book deals with the question of the artificial feeding of infants, and contains a very useful collection of the views of the best-known English authorities upon the subject. The truly terrible ignorance displayed by mothers, especially amongst the poor, upon the subject of infant feeding is answerable for an infant mortality so great as to be appalling."—*Daily Chronicle.*

**DIET IN SICKNESS AND IN HEALTH.** By MRS. ERNEST HART, formerly Student of the Faculty of Medicine of Paris, and of the London School of Medicine for Women. Demy 8vo, blue buckram, gilt, with numerous illustrations, price 3s. 6d.

With an introduction by Sir Henry Thompson, F.R.C.S., M.B. Lond., who says:—"I do not hesitate to express my opinion that the present volume forms a handbook to the subject thus briefly set forth in these few lines, which will not only interest the dietetic student, but offer him, within its modest compass, a more complete epitome thereof than any work which has yet come under my notice."

**A PRACTICAL HANDBOOK OF MIDWIFERY.** By FRANCIS W. N. HAULTAIN, M.D. A practical manual produced in a portable and convenient form for reference, and especially recommended for its compactness, conciseness, and clearness. 18mo., profusely illustrated with original cuts, tables, &c.; about 260 pp., handsomely bound in leather. Price 6s.

"One of the best of its kind, and well fitted to perform the functions its author claims for it."—*Edinburgh Medical Journal.*

**THE ART OF MASSAGE.** By A. CREIGHTON HALE. A complete Text-book of this valuable art. The most practical, simple, and generally useful work published on the subject. With fully illustrated chapters on the Anatomy of the Human Body. Demy 8vo, nearly 200 pp., cloth gilt, profusely illustrated with over 70 special cuts. Price 6s.

"The latest and most complete book we know on Massage."—*Queen.*

**NURSING: ITS THEORY AND PRACTICE.** Being a complete Text-Book of Medical, Surgical, and Monthly Nursing. By PERCY G. LEWIS, M.D., M.R.C.S., L.S.A. Always a standard work on Nursing, it now occupies the position of a classic, being used throughout the world in Training Schools and by Private Students. Eighth Edition. Greatly enlarged and thoroughly revised. Profusely Illustrated. Crown 8vo, cloth, gilt. Price 3s. 6d.

"We warmly recommend it as a nursing manual . . . full of useful hints and information."—*Birmingham Medical Review.*

"Full of interest and instruction . . . cannot fail to assist nurses in their work."—*British Medical Journal.*

**SURGICAL WARD-WORK AND NURSING.** By ALEXANDER MILES, M.D. (Edin.), C.M., F.R.C.S.E. A practical Manual of Clinical Instruction for Nurses and Students. This book will be found especially useful to beginners, to whom its simple description of elementary principles and treatment in surgery will render it invaluable. Demy 8vo, 200 pp., cloth boards, copiously illustrated. Price 3s. 6d.

"Will furnish much-needed guidance to the student and the nurse in the early stages of their apprenticeship."—*Times.*

London: THE SCIENTIFIC PRESS (Limited), 428, Strand, W.C.

### "THE HOSPITAL"

AN INDEPENDENT JOURNAL OF

**The Medical Sciences**

AND

**Hospital Administration,**

WITH WHICH IS ISSUED WEEKLY

A SPECIAL

### SUPPLEMENT FOR NURSES.

PUBLISHED EVERY SATURDAY.

PRICE TWOPENCE.

Matrons, Sisters, or Nurses will find in THE HOSPITAL a chronicle of the latest Nursing News, Practical Articles of the highest value to those who wish to keep themselves abreast with the times, and brightly written articles from Nurses all over the world.

**SUBSCRIPTIONS CAN COMMENCE AT ANY TIME.**

### TERMS OF SUBSCRIPTION.

### WEEKLY ISSUE.

| | Great Britain. | Foreign and Colonial. |
|---|---|---|
| For One Year ... ... | 10s. 6d. ... | 15s. 2d. |
| For Six Months ... | 5s. 3d. ... | 7s. 7d. |
| For Three Months... | 2s. 8d. ... | 3s. 10d. |

### MONTHLY PARTS.

| | Post Free to any Part of the World. |
|---|---|
| For One Year ... ... | 8s. 6d. |
| For Six Months ... ... | 4s. 3d. |
| For Three Months ... ... | 2s. 2d. |

Postal Orders and Cheques should be made payable to "The Scientific Press, Limited."

N.B.—In order to prevent delay, all business communications should be addressed to "THE MANAGER" only, and not to any person individually.

### CHANGE OF ADDRESS.

In event of change of address, notice should be forwarded to the Publishers by the Saturday previous to date of publication, and the old address should also be given.

"THE HOSPITAL" can be obtained of all Booksellers and Newsagents in the United Kingdom, and at all the Railway Stalls, of Messrs. W. H. Smith and Son; and also from the following Agents in the Colonies and Abroad:—

PARIS: Librairie Galignani, 224, Rue de Rivoli.
BERLIN: Messrs. A. Asher and Co.
LEIPSIG: Mr. F. A. Brockhaus.
NEW YORK: The International News Co.
MONTREAL: The Montreal News Co.
TORONTO: The Toronto News Co.
MELBOURNE, SYDNEY, ADELAIDE, and ZEALAND: Messrs. Gordon and Gotch.
INDIA: At all the Railway Bookstalls and Agencies of Messrs. A. H. Wheeler and Co.
SOUTH AFRICA: Messrs. Juta, Cape Town.

PUBLISHING OFFICES: 428, STRAND, LONDON, W.C

**NURSING BOOKS AT FULL DISCOUNT.**

*LIST POST FREE.*

The Theory and Practice of Nursing (Percy Lewis, M.D.), 3/6 for 2/8.
The Nurse's Dictionary (Honnor Morten), 2/- for 1/6.
The Nurse's Case Book, 6d. for 3½d.

NEW EDITION OF DR. CHEADLE'S "ARTIFICIAL FEEDING OF INFANTS."

Now Ready, FOURTH EDITION, Crown 8vo., 5s.

**On the PRINCIPLES and EXACT CONDITIONS to be OBSERVED in the ARTIFICIAL FEEDING of INFANTS; THE PROPERTIES OF ARTIFICIAL FOODS; AND THE DISEASES WHICH ARISE FROM FAULTS OF DIET IN EARLY LIFE.**

By W. B. CHEADLE, M.A., M.D., F.R.C.P.,

Physician to St. Mary's Hospital and Co-Lecturer on Clinical Medicine in St. Mary's Medical School; Consulting Physician to the Hospital for Sick Children, &c.

London: Smith, Elder, and Co., 15, Waterloo Place, S.W.

### ROYAL NATIONAL PENSION FUND FOR NURSES.

*President*—**H.R.H. THE PRINCESS OF WALES.**

*Chairman*—WALTER H. BURNS, Esq. *Deputy-Chairman*—HENRY C. BURDETT, Esq.

**PENSIONS. SICKNESS. ACCIDENT.**

### Total Invested Funds, £250,000.

Nurses are invited to join the Fund on account of the substantial and exceptional advantages which it offers them and which *they cannot obtain elsewhere.* The following are the chief points:—

1. **The Fund is Mutual and essentially Co-operative.**
2. **Easy Payment of Premiums.**
   Nurses can pay their premiums monthly or otherwise as best suits their convenience.
3. **The Fund is open to every Nurse.**
   Nurses can assure for Pensions of any amount commencing at any age.
4. **An Investment and Savings Bank.**
   Those entering under the returnable scale can have their premiums returned to them with compound interest, less a small deduction for working expenses. Interest allowed on deposits.
5. **Additions to Pensions.**
   Besides the Pension entered for, substantial additions thereto may be anticipated in the shape of bonuses.
6. **Sickness and Accident Assurance.**
   Policies are issued in connection with Pension policies assuring 10s., 15s., or 20s. a week in cases of incapacity from work through sickness or accident.

**Full particulars to be obtained from THE SECRETARY,**

ROYAL NATIONAL PENSION FUND FOR NURSES,

28, FINSBURY PAVEMENT, LONDON, E.C.

